# Remedial Technologies for Aniline and Aniline Derivatives Elimination from Wastewater

**DOI:** 10.5696/2156-9614-10.25.200302

**Published:** 2020-01-22

**Authors:** Naveen Kumar Chaturvedi, Surjit Singh Katoch

**Affiliations:** Centre for Energy and Environmental Engineering, National Institute of Technology Hamirpur, Himachal Pradesh, India

**Keywords:** advanced oxidation processes, aniline, 2-methoxyaniline, 4-methoxyaniline, Fenton's reagent

## Abstract

**Background.:**

Aniline and its derivatives are widely used as intermediate chemicals in the pharmaceutical and dye industries and are present in their wastewaters. These chemicals are of concern due to their potential detrimental effects on public health and aquatic species in the environment.

**Objectives.:**

Various available remedial technologies presented in the literature were investigated to determine the most suitable technology for the elimination of aniline and aniline derivatives from waste streams.

**Methods.:**

The related literature was collected electronically from ScienceDirect, Google Scholar, the International Agency for Research on Cancer (IARC), ResearchGate and Wiley Online Library for systematic review. The search terms included ‘aniline’, ‘aniline degradation’, ‘advanced oxidation processes (AOPs)’, ‘aniline derivatives’ and ‘Fenton’s reagent'.

**Discussion.:**

Aniline and its derivatives are a serious issue in the effluents of dye and pharmaceutical industries, but a number of efficient treatment methods using biological, physical and AOPs have been presented in the literature.

**Conclusions.:**

Comparison of the available technologies showed that AOPs were the most cost effective and efficient technologies for eliminating aniline and its derivatives from wastewater.

**Competing interests.:**

The authors declare no competing financial interests.

## Introduction

Aniline is used extensively for producing organic compounds, such as rubber, azo dyes, fuel additives, antioxidants, corrosion inhibitors, pharmaceuticals, antiseptics and pesticides.^1^,^2^ Aniline is considered one of the most toxic aromatic compounds.^3^ Pharmaceutical and dye industry wastewater containing aniline harms aquatic ecosystems due to its incalcitrant structure and high toxicity.^4^ The International Agency for Research on Cancer (IARC) categorizes aniline as a group 2B carcinogenic compound due to its mutagenic and carcinogenic potential.^5^

Two important aniline derivatives are 2-methoxyaniline and 4-methoxyaniline. 2-methoxyaniline, also known as O-anisidine, is a colorless liquid which becomes brownish with air contact and can be absorbed by skin contact, oral ingestion and inhalation.^6–8^ It is an important intermediate in the manufacture of numerous azo and triphenylmethane dyes and pigments, as well as some pharmaceuticals. Its corrosion inhibiting and antioxidant properties make it suitable for use in steel and polymercaptan resins, respectively.^9^ 4-methoxyaniline (or p-anisidine) is a white solid at room temperature with a density of 1.07 g/cm^3^. It is useful in determining food quality, and as dyestuff and pigment intermediates.^10^ The principal physical and chemical properties of 2-methoxyaniline and 4-methoxyaniline are given in Table 1.

**Table 1:** Principal Physical and Chemical Properties of 2- and 4-Methoxyaniline

**Property**	**2-Methoxyaniline**	**4-Methoxyaniline**
Structure	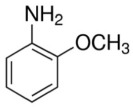	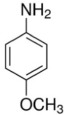
Synonyms	O-anisidine, 2-aminoanisole	P-anisidine, 4-aminoanisole
Physical state	Clear, white liquid – yellow or red-brown when oxidized	White solid, gray or gray-brown when oxidized
Formula	C_7_H_9_NO	C_7_H_9_NO
Melting point (°C)	6.2	57.2
Boiling point (°C)	224	243
Solubility in water	1.5 g/100 ml	less than 1 mg/mL
Density (g/cm^3^)	1.09	1.07

Blood and nerve cells can be damaged by 2-methoxyaniline, leading to cyanosis and suffocation. The chemical has been used experimentally in carcinogenicity investigation and is thought to cause cancer in humans.^11^ 4-methoxyaniline is the most toxic of the three methoxyaniline isomers, releasing nitrogen oxide vapors when heated strongly. The harmful effects of aniline and its derivatives makes these compounds an important target for elimination from waste streams through biological, physical and chemical processes.

In recent studies, advanced oxidation processes (AOPs) have emerged as promising technologies to degrade various pharmaceutical and dye intermediates in wastewaters and reduce their toxicity and refractory nature. Most AOPs are based on the hydroxyl radical (HO•), which has a very high oxidation potential – 2.8 electron volt (eV) *(Table 2).* It can degrade many organic compounds and substantially limit toxicity to aquatic species. Advanced oxidation processes provide several possible ways of generating HO•, which increase their versatility and suit them to the requirements of specific treatments.^12^ Advanced oxidation processes include Fenton oxidation, photo-Fenton oxidation (solar/ultraviolet (UV)) and Fenton-like oxidation.

**Table 2:** Electrochemical Potential Comparison^12^

**Oxidizing agent**	**Oxidation potential (eV)**
Molecular oxygen	1.23
Chlorine dioxide	1.27
Chlorine	1.36
Hypochlorite	1.49
Hydrogen peroxide (H_2_O_2_)	1.78
Ozone	2.08
Atomic oxygen	2.42
**Hydroxyl radical (HO•)**	**2.80**

Abbreviation: eV, electron volt

In the present study, various available remedial technologies presented in the literature were investigated to determine the most suitable technology for the elimination of aniline and aniline derivatives from waste streams.

## Methods

**Figure 1:**
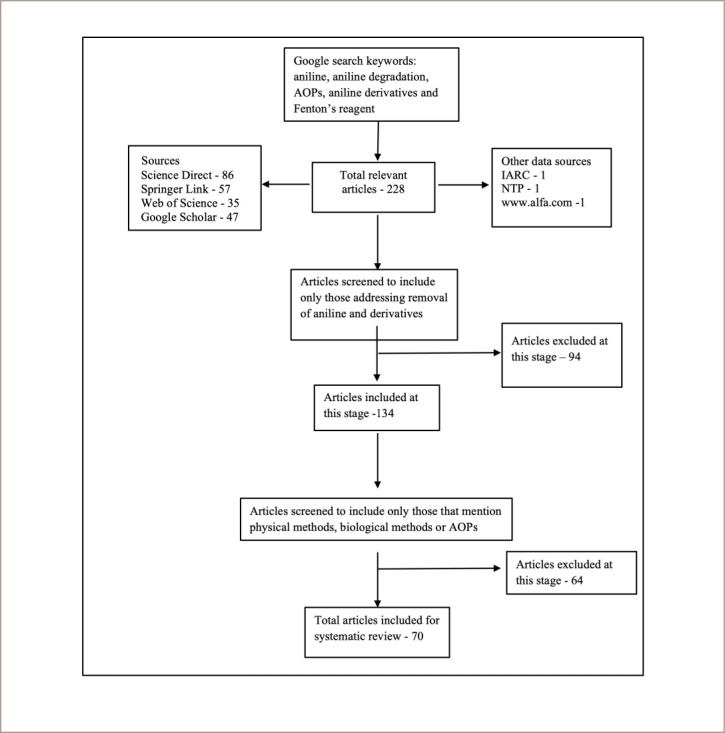
PRISMA flow chart showing the collection, screening, inclusions and exclusions of articles

Literature was collected from ScienceDirect, Google Scholar, SpringerLink, ResearchGate, Wiley Online Library, Web of Science, IARC, National Toxicology Programs (NTP) and the website (www.alfa.com, which provided information on P-anisidine).^10^ Search terms included ‘aniline’, ‘aniline degradation’, ‘AOPs’, ‘aniline derivatives’, 2-methoxyaniline, 4-methoxyaniline and ‘Fenton’s reagent'. A total of 228 articles were collected. One hundred and thirty-four (134) articles were further determined to contain studies explicitly addressing removal of aniline and its derivatives. Articles containing physical methods, biological methods and AOPs for the treatment of aniline derivatives were filtered with special focus on AOPs. Subsequently, articles irrelevant to the present study were eliminated and only 70 articles were included finally and cited in this review. Collection and selection procedures are shown in Figure 1. All 70 articles were reviewed and used in this study to determine the most suitable technique for the degradation of aniline and its derivatives from waste streams. The articles were screened for quality based on the quality of materials used, the use of standard instrumentation and the use of a reference laboratory. The Supplemental Material presents the checklist used for screening the articles for the literature review. Only articles which satisfied at least two parameters (columns) were included for the review. The remainder of the articles were used to collect and present reliable information on anilines, aniline derivatives and AOPs.

**Abbreviations**
*AOPs*: Advanced oxidation processes
*eV*: Electron volt
*H_2_O_2_*: Hydrogen peroxide
*HO*•: Hydroxyl radicals
*IARC*: International Agency for Research on Cancer
*NTP*: National Toxicology Programs
*UV*: Ultraviolet

## Results

The data gathering, screening and extraction revealed three categories of technologies available for treatment of wastewater containing aniline and its derivatives: physical, biological and AOPs. The seventy articles included in the present review are summarized in Table 3.

**Table 3:** Articles Addressing Technologies of Relevance to this Study

**Technologies**	**Sub-category (number of articles)**	**Target compounds (number of articles)**	**Total articles**
Physical	Membrane-based (4) Thermal (1) Adsorption-based (3)	Aniline (5) Other organic compounds (3)	8
Biological	Aerobic (6)	Aniline (4) Other organic compounds (2)	6
AOPs	Fenton (11) Solar/UV-photo Fenton (6) Others: Fenton-like, ultrasound/ozone (US/O3), wet air oxidation, electro-Fenton etc. (18)	Aniline (9) Other organic compounds (26)	35
Combined Technologies	Fenton + biological (2) Fenton + photo-Fenton (3) Other combinations (photo-Fenton + biological, Fenton + Fenton-like, electro-Fenton + fluidized bed Fenton, electro-Fenton + peroxi-coagulation, photo-catalysis + ozonation) (5)	Aniline/mixture of other compounds (10)	10
**Other included articles**			
	
2-methoxyaniline (4) 4-methoxyaniline (2) IARC and NTP 2016 (2) Others relevant articles providing information on AOP reactions (3)			11
		Total Number of Articles	70

### Physical technologies

Several physical treatment technologies, such as adsorption, thermal incineration and membrane filtration have been used to eliminate aniline and aniline derivatives from wastewater. Multi-walled carbon nanotubes have been efficiently used to adsorb aniline and its derivatives from aqueous solution.^13^,^14^ Thermal incineration of aniline was also carried out in some studies, but incineration involves heavy fuel consumption and incomplete combustion can lead to air pollution.^15^ Some studies used a mixed bed reactor with a liquid emulsion membrane and were able to remove 98.53% of the aniline present in wastewater.^16^ Aqueous solutions containing aniline have reportedly been treated by chemical desorption and permeation using a silicone membrane.^17^,^18^

### Biological technologies

A variety of microorganisms have been applied to remove aniline from wastewater via biological treatment methods. Aniline can be successfully removed by microorganisms such as Dietzia natronolimnaea, Pseudomonas *sp.*, Delftia *sp.* and Pigmentiphaga daeguensis.^19–23^ Biological methods are efficient and ecofriendly, as they use natural pathways to achieve the required wastewater quality, but in the case of incalcitrant organic compounds, like aniline and its derivatives which can be toxic to microorganisms, they are impractical.^24^,^25^ Pharmaceutical wastewater contains a variety of harmful compounds and treatment to the required effluent standards is difficult. The main problem with biological treatment is that it is difficult to grow cultures, taking up to a year, and to maintain them in pure form on a large scale. In addition, it takes 15 to 20 days to oxidize the organic contaminants. Daily monitoring is required to maintain good environmental conditions for microorganism growth.

### Advanced oxidation processes

Advanced oxidation processes exploit the high oxidative power of HO• to remove organic contaminants and have been successfully used to eliminate refractory organic pollutants from wastewater. They can degrade organic contaminants by oxidation via chemical and photochemical processes in the presence of a catalyst.^26^,^27^ Advanced oxidation processes depend on the generation of powerful oxidants to remove organic species.^28–30^ Most available AOPs are HO• based, but some are based on oxidizing agents like chlorine or sulfate radicals.^30^Hydroxyl radical's oxidation potential is 2.80 eV, which exceeds most other chemical agents *(Table 1),* and its rate constants are higher than those of other processes like ozonation. Hydroxyl radical is highly reactive and unstable in nature and must be produced constantly in situ by chemical reaction.^31–33^

Advanced oxidation processes include a wide variety of treatment processes, such as Fenton's oxidation, Fenton-like oxidation, photo-Fenton oxidation, solar photo-Fenton's oxidation, titanium dioxide-assisted photolysis and electro-Fenton oxidation. The mechanism and reaction kinetics of Fenton's (ferrous ion (Fe^2+^)/hydrogen peroxide (H_2_O_2_)) and Fenton-like oxidation (ferric ion (Fe^3+^)/H_2_O_2_) have been widely researched and will be discussed later in this study.^34–36^ Several aniline derivatives have been treated and degraded successfully using AOPs. Degradation of p-nitroaniline, p-aminophenol and acetanilide has been studied using solar photo-Fenton and UV photo-Fenton treatment, establishing that both methods were more beneficial than the basic Fenton process due to their greater oxidation ability, wider pH tolerance and low Fe^2+^ requirement.^37^,^38^

In other work, aniline wastewater was treated by both biological and photo-Fenton oxidation separately and then conjointly with biological and photo-Fenton degradation.^39^ The effective pH range was 3–4 and photo-Fenton oxidation successfully enhanced the biodegradation of aniline. The maximum aniline degradation attained was 94% with the combined biological and photo-Fenton processes. Aniline was oxidized and 2-nitroaniline degraded using the photo-Fenton process, and the Fenton and photo-Fenton processes, where aniline removal efficiency was 84.14% and 93.8%, respectively. 40,41

Many studies have demonstrated the effectiveness of Fenton's reagent (Fe^2+^/H_2_O_2_) in degrading toxic organic compounds in wastewater.^39^,^42^,^43^ The aniline removal efficiency by Fenton's reagent in a fluidized bed increased until H_2_O_2_ concentration reached a threshold value, above which no further removal was observed. Although the electro-Fenton process is more efficient than the fluidized bed version, H_2_O_2_ depletion is much higher in the former, making the fluidized bed more economic.^1^ Aniline oxidation by ozonation and titanium dioxide-assisted photocatalysis showed increased total organic carbon removal when aniline was pretreated with ozone.^44^,^45^ Remedial technologies for aniline and its derivatives are summarized in Table 4.

**Table 4:** Remedial Technologies for Aniline and Aniline Derivative

**Technologies**	**Process/microorganism**	**Elimination achieved**	**Merits**	**Demerits**
Physical	Micellar enhanced ultrafiltration^46^ Adsorption on cobalt-supported pumice^47^ Liquid emulsion membrane^16^ Thermal incineration^15^	70% removal of aniline Process found to be efficient 98.53% removal of aniline Complete removal of aniline	Physical processes are fast and efficient in removal of aniline from wastewater	Membrane fouling Heavy fuel consumption High energy demand Secondary pollution
Biological	Delftia sp.^19^ Dietzia natronolimnaea^20^ Pseudomonas sp.^22^ Pigmentiphaga daeguensis^23^	Complete removal of aniline in 22 hours 87% removal in 120 hours Complete removal in 24 hours Complete removal of aniline in 15 hours	Biological processes are efficient and ecofriendly in eliminating specific organic compounds by specific microorganisms	Difficulty degrading toxic organic compounds Slow process and high maintenance Bad odor and fly nuisance
AOPs	Photo Fenton oxidation and biological oxidation^39^ Reaction with ozone in presence of zero valent zinc^48^ Removal by ultrasound/ozone combination^49^ Photo Fenton and Fenton oxidation^50^	94% removal of aniline by combined process Complete aniline removal in 25 minutes Near complete removal of aniline after 30 minutes 90% and 82% removal of 3-aminopyridine with iron(III) sulfate and laterite soil extract, respectively	Easy to operate Economical and ecofriendly process with faster rate of degradation Can degrade non-selectively almost all organic compounds Variety of process available	pH dependence Sludge formation Complex reaction chemistry

The most cost-effective, promising and easily operable AOP is Fenton's oxidation. It has been applied to treat harmful organic compounds since the 1960s.^51^,^52^ Fenton's reagents are comprised of H_2_O_2_ and Fe^2+^. They have been used to eliminate organic and inorganic species including hypochlorite, sulfite, nitrite, chlorine and cyanide.^53^,^54^

The key oxidizing agent in Fenton's oxidation is HO•, which arises from the reaction of Fe^2+^ and H_2_O_2_ in an acidic medium and is shown in Equation 1:

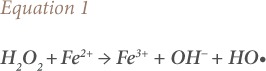
where, OH^−^ is hydroxyl ion Hydroxyl radical can oxidize organic compounds due to its 2.8 eV oxidation potential.^53^,^55^,^56^

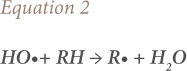
where, RH is the organic compound, R• is an organic radical, and H_2_O is water.

The simplified reaction during Fenton's treatment is represented by Equation 3.^34^

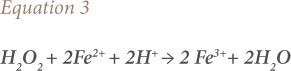
where, H^+^ indicates hydrogen ions and H_2_O is water.

Equation 3 demonstrates that an acidic environment is crucial during Fenton's oxidation, to increase the HO• concentration to obtain maximum degradation of target organic species. A pH level close to 3.0 is optimum in Fenton's oxidation.^57^

Fenton's reactions using catalysts other than Fe^2+^ are called Fenton-like reactions, and are shown in Equation 4.^58^

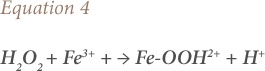
where, Fe-OOH^2+^ is ferrous hydroxide (in aqueous medium) and H^+^ represents hydrogen ions. As Fe^3+^ react with H_2_O_2_ in place of Fe^2+^ (*Equation 4*) the Fe-OOH^2+^ dissociates into peroxide radicals (HO_2_•) and ferrous ions, shown in Equation 5:

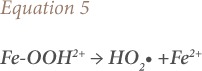
where, Fe-OOH^2+^ is ferrous hydroxide (in aqueous medium) and HO_2_• indicates peroxide radicals

Fe^2+^ produced in this way (*Equation 5*) reacts again with H_2_O_2_, yielding HO•, which degrades organic compounds present in the water following Equations 6 and 7.

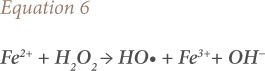


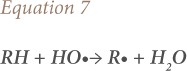
where, OH^−^ is hydroxyl ions, RH is the organic compound and R• is an organic radical.

Fenton oxidation in the presence of light, e.g., solar or UV radiation, is termed photo-Fenton oxidation.^59^ The illumination increases the amount of HO• generated expressed in Equations 8 and 9.^60^

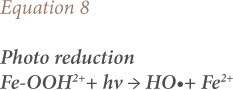


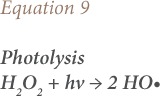
where, *hv* is radiation and Fe-OOH^2+^ is ferrous hydroxide (aqueous medium).

There are several key parameters which influence the effectiveness of AOPs. Wastewater pH can significantly enhance AOP effectiveness.^61^ Oxidation by AOPs is slower in alkaline conditions, while acidic media can be effective in making degradation faster.^62–65^ The concentration ratio of H_2_O_2_ to Fe^2+^ is key to pollutant removal in AOPs based on Fenton's reagent. Increasing the Fe^2+^ concentration results in increased HO• concentration, which improves the degradation efficiency.^66^ If the Fe^2+^ concentration exceeds the optimum, pollutant and chemical oxygen demand degradation is inhibited, as the Fe^2+^ starts to absorb the free HO•.^67^ The higher the initial pollutant concentration, the higher the consumption of H_2_O_2_, while the catalytic reaction between Fe^2+^ and H_2_O_2_ is also hindered, thereby reducing the reaction efficiency.^68^

## Discussion

Aniline and its derivatives have been treated and eliminated by various technologies including physical, biological and AOPs. Although all of these technologies were capable of eliminating aniline and its derivatives from waste streams, they have several limitations. The physical treatment processes were found to be efficient and fast, but their disadvantages include creation of secondary pollution in case of thermal incineration and high maintenance costs due to energy. In membrane filtration processes the regular cleaning of the membrane by backwashing requires energy, thereby increasing costs. In addition, fouling of membranes over time is a significant disadvantage of this technology. Biological processes are the most eco-friendly techniques and were found to be effective in the elimination of several organic compounds. However, the effectiveness of biological processes depends on the type of substrate available to be acted upon by microbes. Therefore, in the case of incalcitrant and toxic compounds like aniline and its derivatives, biological processes are impracticable. In addition, biological process limitations include slower elimination, and difficulties with maintenance and culture growth in pure form. Because of these and several other problems, chemical pretreatment by AOPs should be considered. This may enhance biodegradability as toxins are removed from wastewater prior to biological treatment.^69^,^70^The AOPs were capable of converting organic compounds, irrespective of their origin, into simpler molecules and sometimes complete elimination into carbon dioxide and water. Advanced oxidation processes range from simple processes like classic Fenton's oxidation to complex processes like electro-Fenton and UV/titanium dioxide-based photolysis. Hydroxyl radicals are the key species applied in almost all AOPs to degrade organic contaminants in wastewater. Advanced oxidation processes are the fastest, most economical and effective treatment technologies available in the literature, but have limitations, including sludge formation, pH dependence and maintenance and complex reaction chemistry.

## Conclusions

The present review demonstrated that aniline and its derivatives, including 2-methoxyaniline and 4-methoxyaniline, are commonly produced and discharged in waste streams. Due to their toxicity, carcinogenicity and adverse effects on human and aquatic species, wastewater containing these compounds must be treated prior to disposal. Several treatment technologies were identified in the literature to eliminate these compounds from wastewater. Physical and biological treatment processes were found to be effective, but have many limitations such as high energy demands, secondary pollution, slower elimination rate, cleaning and maintenance. These limitations can be easily overcome by AOPs as they have been proven to be more cost effective and efficient in removing aniline and other organic compounds from wastewater. Almost all AOPs involving HO• as the oxidizing agent work best in a pH range of 3–4 for organic contaminant removal. Hydroxyl radicals non-selectively degrade almost all organic contaminants to carbon dioxide and water, or into biodegradable forms on occasion. In some cases, AOPs were engaged conjointly with biological processes resulting in faster and efficient degradation than standalone processes, thereby making them a successful pre-treatment option for incalcitrant and toxic organic compounds to be subsequently treated by biological processes. Advanced oxidation processes like photo-Fenton oxidation with UV degrades organic contaminants more efficiently than Fenton or solar-Fenton oxidation. In addition, the solar-Fenton process has higher oxidation ability across a wider pH range with lower Fe^2+^ usage than the classic Fenton process. Finally, the study suggested that AOPs are the most suitable remedial measure to eliminate organic compounds and can be applied to wastewater containing aniline and aniline derivatives (2-methoxyaniline and 4-methoxyaniline).
