# Supplementary material for: Remedial Technologies for Aniline and Aniline Derivatives Elimination from Wastewater
Source: J Health Pollut. 2020 Jan 22;10(25):200302. doi: 10.5696/2156-9614-10.25.200302 (PMC7058138; doi:10.5696/2156-9614-10.25.200302)
Supplement: Supplementary file 1 [file Chaturvedi_Supplemental_Material.docx]

**Supplemental Material**

Naveen Kumar Chaturvedi & Surjit Singh Katoch

**Checklist for Inclusion of Articles in Study**

| Author | Technology used | | | Target compound | | |
| --- | --- | --- | --- | --- | --- | --- |
|  | Physical | Biological | AOPs | Aniline | Aniline derivatives | Other compounds |
| Anotoi *et al.***^1^** |  |  | ✓ | ✓ |  |  |
| Brillas and Casado**^2^** |  |  | ✓ | ✓ |  |  |
| Jagtap and Ramaswamy**^3^** |  |  | ✓ | ✓ |  |  |
| Sun *et al.***^4^** |  |  | ✓ |  | ✓ |  |
| Sharma *et al.***^12^** |  |  | ✓ | ✓ | ✓ | ✓ |
| Nxumalo *et al.***^13^** | ✓ |  |  | ✓ |  |  |
| Al-Johani and Salam**^14^** | ✓ |  |  | ✓ |  |  |
| Xie *et al.***^15^** | ✓ |  |  | ✓ |  |  |
| Datta *et al.***^16^** | ✓ |  |  | ✓ |  |  |
| Ferreira *et al.***^17^** | ✓ |  |  | ✓ |  |  |
| Sawai *et al.***^18^** | ✓ |  |  | ✓ |  | ✓ |
| Xiao *et al.***^19^** |  | ✓ |  | ✓ |  |  |
| Jin *et al.***^20^** |  | ✓ |  | ✓ |  |  |
| Liu *et al.***^21^** |  | ✓ |  | ✓ |  |  |
| Jiang *et al.***^22^** |  | ✓ |  | ✓ |  |  |
| Huang *et al.***^23^** |  | ✓ |  | ✓ |  |  |
| Gotvajn and Zagorc**^24^** |  | ✓ | ✓ |  |  |  |
| Padoley *et al.***^25^** | ✓ | ✓ | ✓ |  | ✓ | ✓ |
| Oliveros *et al.***^26^** |  |  | ✓ | ✓ | ✓ | ✓ |
| Kavitha *et al.***^27^** |  |  | ✓ |  |  | ✓ |
| Bolton *et al.***^28,29^** |  |  | ✓ |  |  | ✓ |
| Miklos *et al.***^30^** |  |  | ✓ |  | ✓ | ✓ |
| Esplugas *et al.***^32^** |  |  | ✓ |  |  | ✓ |
| Zhu *et al.***^33^** |  |  | ✓ |  |  | ✓ |
| Sun *et al.***^37^** |  |  | ✓ |  | ✓ |  |
| Sheikh *et al.***^38^** |  |  | ✓ | ✓ | ✓ | ✓ |
| Liu *et al.***^39^** |  | ✓ | ✓ | ✓ |  |  |
| Azizi *et al.***^40^** |  |  | ✓ | ✓ |  |  |
| Amritha and Manu**^41^** |  |  | ✓ |  | ✓ |  |
| Andreozzi *et al.***^42^** |  |  | ✓ |  |  | ✓ |
| Mingyu *et al.***^43^** |  |  | ✓ |  |  | ✓ |
| Sanchez *et al.***^44^** |  |  | ✓ | ✓ |  |  |
| Orge *et al.***^45^** |  |  | ✓ | ✓ |  |  |
| Tanhaei *et al.***^46^** | ✓ |  |  | ✓ |  | ✓ |
| Bardakci *et al.***^47^** | ✓ |  |  |  | ✓ |  |
| Zhang *et al.***^48^** |  |  | ✓ | ✓ |  |  |
| Song *et al.***^49^** |  |  | ✓ | ✓ |  |  |
| Karale *et al.***^50^** |  |  | ✓ |  | ✓ | ✓ |
| Huang *et al.***^51^** |  |  | ✓ |  |  | ✓ |
| Matavos-Aramyan and Moussavi**^52^** |  |  | ✓ |  |  | ✓ |
| Li *et al.***^65^** |  |  | ✓ |  |  | ✓ |
| Manu and Mahamood**^67^** |  |  | ✓ |  |  | ✓ |
| Manu *et al.***^68^** |  |  | ✓ |  |  | ✓ |
| Gotvajn *et al.***^69^** |  | ✓ | ✓ |  | ✓ | ✓ |
| Padoley *et al.***^70^** |  | ✓ | ✓ |  |  | ✓ |

- Only articles which satisfied at least two parameters (columns) were included for the review. The remainder were used to collect and present reliable information on anilines, aniline derivatives and AOPs.
- Articles reference no. 5-11 were used to introduce aniline derivatives: 2- and 4-methoxyanilines and their uses and adverse effects on the environment.
- Articles reference no. 31, 34-36, 53-64 and 66 were included for background information on AOPs and the chemical reactions involved in Fenton’s oxidation and were cited where appropriate.
